# Upon the Folly and Danger of Hypotheses, with Remarks on Mr. Walker's Innate Principle of Small-Pox

**Published:** 1814-12

**Authors:** James Woodham

**Affiliations:** Surgeon, And Assistant-Surgeon and Apothecary to the Central Dispensary. West Smithfield


### For the Medical and Physical Journal.

### Upon the Folly and Danger of Hypotheses, with Remarks on Mr. Walker's innate Principle of Small-pox; by Mr. WOODHAM.

"Quicquid ex phœnomenis non deducitur, HYPOTHESIS vocanda est."

NEWTON.

WITHOUT feeling any Baconian honour, to speak in the language of a Northern critic, at hypotheses, I own I am so much of a Baconian as to entertain for them a strong dislike. Why I dislike them, is not merely because I consider it a waste of time and talents to be employed on subjects which cannot for the most part be verified either by observation or experiment, but that they are frequently productive of the most serious mischief. I will say nothing of the evils that have arisen from the treatment formerly adopted in small-pox, when the wretched victims of an absurd hypothesis, confined in close apartments, sweltering under a load of bed-clothes, and immersed in the fumes of their own perspiration, welcomed death as a kind release from both the disease and the doctor; or of the tedious and debilitating courses of mercury employed, from an equally absurd hypothesis, for the cure of the venereal disease, when rivers of saliva, if I may use the expression, were made to flow from the swollen and ulcerated mouths of the unhappy sufferers, so that, on their protracted recovery, they were left in doubt which most to execrate—the disorder or the remedy. I will pass these, and come to our own times. The spasm and atony of Cullen, whose name I repeat with reverence as my first instructor in medicine, and still more the accumulated excitability of his rival Brown, have sent numbers to an untimely grave. Fearful of debility, and from an endeavour to relax the imaginary spasm, patients labouring under fever were treated by the Cullenians principally by the exhibition of those medicines which are said to determine to the surface; bleeding and purging, the only effectual remedies, particularly in fevers occurring in warm climates, were almost entirely neglected, or sparingly used, and the disease was suffered to pursue its course, till it was removed by death, or a slow recovery. By the Brunonians, much greater evils were occasioned. To remove the accumulated excitability, the proximate cause they said of fever, not only venesection and purging were neglected, but even the feeble means of sweating. They despised and discarded these, and poured into their patient's stomachs the whole tribe of their diffusible stimuli, brandy, wine, æther, opium, &c. till they found that in exhausting their excitability they had sacrificed their patients.

 These

These observations were suggested by the perusal of a paper in the last Number of your Journal, on Vaccination, or, more correctly speaking, on the vaccine and variolous diseases, by Mr. Walker, of Oxford, the gentleman, I believe, to whom the philosophical world is indebted for some experiments on frigorific mixtures. As in that paper there are some opinions which I conceive to be not merely hypothetical, but contradictory, I shall, with your permission, take the liberty of making on them a few remarks.

In the early part of the paper, Mr. Walker observes, "I consider every person as having the essence or innate principle of the small-pox originally in the system, which remains *dormant*, unless matured and put into action by an exciting cause, of which *infection* is the usual one, and to which every individual is so liable to be exposed, that no one has a reasonable expectation of escaping it." If by essence or innate principle, Mr. Walker had meant only a susceptibility to be acted on by the causes, whatever they be, which produce small-pox, no one, I believe, would differ from him; but then we have also originally in the system the essence or innate principle of fever, sore-throat, rheumatism, and every disease to which we are liable. He continues, "Whenever the essence or innate principle of the disease is by any means put into action, it assimilates to its own nature a certain portion of the fluids, circulating in the system more or less according to the grossness or purity of the habit, and throws out or expels them. If the whole of the original essence be expelled, or the system entirely purified of the disposition, which is ordinarily the case, the person will never be subject to a second attack. The more complete the ferment or commotion in the system may have been at the time of the disease, the greater will be the probability of the whole having been thrown off, and *vice versa*." That persons having had the small-pox, either in what is termed the natural way, or by inoculation, are not generally liable to have the disease a second time, is an established fact; but that this is owing to the innate principle, from being excited into action, assimilating to itself a certain portion of the fluids, more or less according to the habit's grossness or purity, its being afterwards wholly expelled; and that the more complete the ferment or commotion is, the greater the probability that the whole is thrown off, and the contrary, is an hypothetical assertion incapable of the smallest proof. As well might it be said that this essence or innate principle is an alkali; and that the exciting cause is an acid, that coming in contact, an effervescence or commotion takes place in the lymph or blood, and a neutral salt is formed, formed, which being wholly expelled from the system, leaves the person ever after secure from the disease.

He goes on to observe, "It is well known, that the smallest portion possible of the matter* of the *small-pox*, or *cow-pox*, in an active state, communicated to the absorbent or inhaling vessels, is sufficient to produce either of these diseases, &c." If such be the fact, as it unquestionably is, and if matter be the *essence* which every one has originally in his system of the *small-pox*, then this matter, taken up by the absorbents, not merely as is usually understood, produces certain motions in the system, which are followed by the formation of matter similar to itself, or, as Mr. Walker terms it, assimilates a certain portion of the fluids to its own nature; but a previous process takes place, it excites the essence or innate principle into action, that is, it excites a portion of itself into action. It is, therefore, according to circumstances, both agent and patient, cause and effect, the excitement to action, and the recipient of that excitement.

Such are the inconsistencies and contradictions to which hypotheses lead. Their age, however, is hastening to a close. The inductive philosophy, of which so pure an example has been given by Dr. Hamilton of Edinburgh, is daily gaining ground; and while the spasm of Cullen, and the lentor of Boerhaave, quietly repose in the same tomb with the commotions and ebullitions of the immortal Sydenham, we cannot but regret that those illustrious characters should have indulged in such puerile conceits, or supposed that their fame was to rest on any other basis than the solid and durable one of observation and experience.

JAMES WOODHAM, Surgeon,

*And Assistant-Surgeon and Apothecary to the Central Dispensary.*

* By the term *matter*, I mean the *essence* of the disease, whether it be in "a *serous* or *purulent state.*"

