# Errata

**Published:** 1815-01

**Authors:** 


					-In Mr.
Woodhan/s paper, for Baconian honour, read Baconian horror.

				

